# Stem-like plasticity and heterogeneity of circulating tumor cells: current status and prospect challenges in liver cancer

**DOI:** 10.18632/oncotarget.12569

**Published:** 2016-10-11

**Authors:** Margherita Correnti, Chiara Raggi

**Affiliations:** ^1^ Center for Autoimmune Liver Diseases, Humanitas Clinical and Research Center, Rozzano, Italy

**Keywords:** circulating tumor cells, primary liver cancer, cancer stem cells

## Abstract

Poor prognosis and high recurrence remain leading causes of primary liver cancerassociated mortality. The spread of circulating tumor cells (CTCs) in the blood plays a major role in the initiation of metastasis and tumor recurrence after surgery. Nevertheless, only a subset of CTCs can survive, migrate to distant sites and establish secondary tumors. Consistent with cancer stem cell (CSC) hypothesis, stem-like CTCs might represent a potential source for cancer relapse and distant metastasis. Thus, identification of stem-like metastasis-initiating CTC-subset may provide useful clinically prognostic information. This review will emphasize the most relevant findings of CTCs in the context of stem-like biology associated to liver carcinogenesis. In this view, the emerging field of stem-like CTCs may deliver substantial contribution in liver cancer field in order to move to personalized approaches for diagnosis, prognosis and therapy.

## STEMNESS ASPECTS OF PRIMARY LIVER CANCER

Primary liver cancer (PLC) is one of the most common cancers worldwide and second leading cause of cancer-related mortality [1, 2]. Primary liver tumors are grossly classified in hepatocellular carcinoma (HCC) and cholangiocarcinoma (CCA) [1, 3–5]. HCC accounts for approximately 90% of all PLC [1, 3], while CCA, a rare tumor but with an increasing global incidence, is the second most common form and accounts for about 5% of all PLC [3–5]. Although liver transplantation, surgical resection and locoregional therapies are effective therapeutic options at early phases, the majority of PLC patients still present unresectable stages of the disease, for which curative options are limited [2]. Currently, the standard-of-care treatment for advanced liver cancer is limited to systemic chemotherapy [1, 5–9]. Unfortunately, results of ongoing clinical trials are discouraging overall and they highlight the urgent need for innovative treatment approaches [10–17]. Additionally, traditional clinicopathological parameters such as tumor morphology, histopathological features and tumor staging system offer limited information for predicting postoperative recurrence [1, 5]. Thus moving to prognostic models for a personalized approach is essential.

The phenotypic overlap between HCC and CCA has been shown to comprise a continuous liver cancer spectrum [18–20]. In this respect, new exciting insights into tumor biology have been recently provided since the discovery of cancer stem cells (CSCs) in many human solid tumors including liver cancer [2, 21–29]. CSCs represent a cellular subset within a tumor endowed with stem-like properties such as the ability for self-renewal and differentiation as well as the resistance to drugs [29, 30]. More importantly, CSCs are thought to be responsible for tumor initiation, recurrence and metastasis showing reduced sensitivity to chemotherapy compared to bulk tumor cells [21, 22, 29, 31]. Indeed growing evidence confirmed that PLC-CSCs are characterized by higher resistance to commonly used therapeutic drugs (e.g. cisplatin, 5-fluorouracil and Sorafenib) [32–39].

It is now becoming accepted that CSCs represent a dynamic and very plastic population [40]. Truly, strength of their plasticity has been certainly given by the aberrant activation of a latent embryonic program (known as the epithelial-mesenchymal transition, EMT) that can generate undifferentiated cancer cells endow with stemness traits [30, 41, 42]. Therefore, EMT-process provides a ready source of CSC-state by enabling the dedifferentiation of epithelial cells within tumor bulk [30, 41, 43–45]. Since CSCs are defined as dynamic entities, accumulating evidence has revealed that tumor microenvironment, defined by signals and cellular interactions arising in the CSC-associated niche [22, 46–48], is involved in regulation and maintenance of stem-like features. Notably, stroma-component of CSC-niche may secrete signaling factors involved in the activation of EMT-programs.

During the last decade there has been a great quantity of studies aimed to identify liver CSCs and several attempts have been made to enrich CSCs in hepatic tumors. Common strategies for PLC-CSC enrichment, varied from the widely used classical antigenic approaches based on the identification of surface stem-like markers (e.g. CD133 [31, 49, 50], CD44 [51], OV6 [52], CD90 [53, 54], epithelial cell adhesion molecule (EpCAM) [55, 56], CD13 [57], CD24 [58], CD47 [59]) to functional methodologies including Side Population analysis [23, 60], Aldefluor assay [61] and sphere formation coupled with serial sphere passaging [62, 63]. In all diverse published studies, enriched PLC stem-like subsets were tested in immune-deficient mice for the *in vivo* tumorigenic potential [23, 31, 49–63] (Table 1). More interestingly only those putative PLC stem-like subpopulation capable to initiate tumor development at low cell numbers, were further tested for ‘self-renewal’ capacity in serial tumor transplantations and molecularly for presence of hepatic stemness-related pathways (e.g. developmental signaling and transcription factors, epigenetic regulation including specific miRNAs) [23, 62, 64–94] (Table 2 and reviewed in [47]). Although a clear phenotypic and functional heterogeneity among the identified liver stem-like cancer subsets [2, 47], PLC-CSCs’ enrichment by different approaches suggests a possible overlapping within several tumorigenic populations. Hence, a combinatorial strategy might be a valid alternative to isolate a better-defined stem-like subset.

**Table 1:** Isolation of Liver CSCs

Methods	Frequency (%)	Minimal Cell Number for Tumor Initiation	References
**Cell Surface Markers**			
CD133	0-65	1000	[31, 49, 50]
CD44	0.1-1.9	100	[51]
OV6	0.2-3	5000	[52]
CD90	0-2.5	500	[53, 54]
EpCAM	0-99	200	[55, 56]
CD13	0.5-1.6	500	[57]
CD24	0.5-97	500	[58]
CD47	9.3-81	500	[59]
**Functional Assays**			
Side Population	0.25-1.2	100	[23, 60]
Aldefluor	1-55	500	[61]
Sphere Culture	1-60	100	[62, 63]

Summary of different methods used to isolate liver CSCs. Frequency of isolated CSCs and their minimal cell number required for tumor initiation are indicated for each assay or marker used, with the respective references.

**Table 2:** Stemness-Related Signalings in Liver CSCs

**Developmental Pathways and Transcription Factors**		**References**
Wnt	Embryonic development, cell fate determination, cell proliferation	[64–67]
Notch	Cell-fate decision during embryonic development and adult life, regulator of self-renewing tissues	[68–73]
Hedgehog	Key regulator of embryonic development	[74–76]
TGF-β	Stem cell renewal and lineage specification; however has a controversial role on HCC genesis as a results from its effect on tumor microenvironment	[77–81]
Bmi1	Stem cell factor, proto-oncogene	[64, 82, 83]
Sall4	Key factors for maintenance of pluripotency and self renewal of embryonic stem cell potentially through the interaction with Oct4,Sox2, Nanog	[84–86]
**Epigenetic Regulators**		**References**
HDAC3	Histone acetylation/deacetylation alters chromosome structure, affects transcription factor access to DNA thus impacts cell cycle progression and developmental events	[87, 88]
DNMT1 DNMT3b	DNA methylation patterns are essential for embryonic stem cell maintenance, mammalian development and normal functioning of adult organism	[23, 62, 88, 89]
**miRNAs**		**References**
miR-142-3p miR-130b	Regulation of self-renewal capability of CD133+ cells	[90, 91]
miR-Let7	Inhibited by Lin28, marker of human embryonic stem cells	[92, 93]
miR-181	Regulation of Wnt/β-catenin pathway	[94]

Summary of stemness-related signalings with a role in liver CSC biology. Functions and relative references are indicated for each pathway, transcription factor, epigenetic regulator or miRNA.

**Table 3:** Summary of CTC studies in PLC patients

N° of Patients		Platform	Method for Detection	CTC Phenotype	CTC Number/Frequency	Method for Characterization	Correlation/Outcome	References
**HCC patients**								
36 PLC		na	Ficoll gradient + CD45 depletion and CD90 magnetic selection	CD45- CD90+ CD44+	0-6.9 %	*In vivo* tumorigenic assay, qRT-PCR	Tumor size	[53]
34 PLC		na	Flow cytometry	CD45- CD90+	0-1.2 %	*In vivo* tumorigenic assay	na	[54]
59		CellSearch System	Positive immuno-magnetic selection	DAPI+ CD45- CK+ EpCAM+	0-5 / 7.5mL	na	OS, BCLC stage, vascular invasion, AFP level	[108]
123	87	CellSearch System	Positive immuno-magnetic selection	DAPI+ CD45- CK+ EpCAM+	1-34 / 7.5mL	Immunofluorescence	Recurrence, TTR, AFP level, vascular invasion, Edmondson stage	[109]
	30	RosetteSep Human CD45 Depletion Cocktail	Negative immuno-magnetic selection	CD45-	1-34 / 7.5mL	qRT-PCR assays	Recurrence, TTR, AFP level, vascular invasion, Edmondson stage	
	6	na	Magnetic-activated cell sorting	CD45- EpCAM+	1-34 / 7.5mL	*In vivo* tumorigenic assay	Recurrence, TTR, AFP level, vascular invasion, Edmondson stage	
21	1	CellSearch System	Positive immuno-magnetic selection	DAPI+ CD45- CK+ EpCAM+	0.14 ± 0.65 / 7.5mL	na	na	[106]
	19	IsoFlux	Ficoll gradient + EpCAM-based magnetic selection on microfluidic device	Hoecht 33342+ CD45- CK+ EpCAM+	127.52 ± 295.15 / 7.5mL	Immunofluorescence	na	
20		CellSearch System	Positive immuno-magnetic selection	DAPI+ CD45- CK+ EpCAM+	0-79 / 7.5mL	Genome sequencing	AFP level, vascular invasion	[131]
85		AutoMACS Pro Separator	Ficoll gradient + ASGPR-based magnetic selection	DAPI+ CD45- ASGPR+ HepPar1+	19 ± 24 / 5mL	Immunofluorescence, FISH, qRT-PCR	Portal vein thrombus, Milan criteria, Edmondson/TNM stage, tumor size	[128]
109		na	Negative immuno-magnetic selection	DAPI+ CD45- CK+ pERK/Akt+	52 ± 23 / 5mL	Immunofluorescence	PFS, therapeutic response to Sorafenib	[113]
299		RosetteSep Human CD45 Depletion Cocktail	Negative immuno-magnetic selection	CD45- EpCAM+	41.2-54.5%	qRT-PCR for EpCAM	Treatment response, TTR, recurrence	[115]
60		MiniMACS Separator	Ficoll gradient + ASGPR-based magnetic selection	HSA+ DAPI+ CD45- ASGPR+	na	Immunofluorescence	Recurrence, portal vein thrombus, Milan criteria, Edmondson/TNM stage, tumor size	[135]
11		RosetteSep Human CD45 Depletion Cocktail	Negative immuno-magnetic selection	DAPI+ CD45- panCK/EpCAM/ASGPR1+ Ncadherin/vimentin+	5-275 CK+ / 1000 PBMC	Immunofluorescence	TTP, cirrhosis	[146]
82		na	Ficoll gradient	CD45- CD90+ CD44+	0-0.02%	Flow cytometry	Tumor size, TNM stage, recurrence	[149]
96		na	Ficoll gradient	Lin28B+	na	qRT-PCR for Lin28B	Recurrence, tumor grade, tumor size, AJCC/BCLC stage,	[154]
2		na	Flow cytometry	CD45- ICAM1+	0.3 ± 0.02 %	Sphere assay, *in vivo* tumorigenic assay	OS, portal vein thrombus, ascites	[155]
44		ISET	Cell size	β-catenin mutated	na	Nested PCR for β-catenin	Tumor diffusion, portal tumor thrombosis, survival, Child-Pugh class, AFP level	[172]
**CCA patients**								
36 PLC		na	Ficoll gradient + CD45 depletion and CD90 magnetic selection	CD45- CD90+ CD44+	0-6.9 %	qRT-PCR	Tumor size	[53]
34 PLC		na	Flow cytometry	CD45- CD90+	0-1.2 %	*In vivo* tumorigenic assay	na	[54]
13		CellSearch System	Positive immuno-magnetic selection	DAPI+ CD45- CK+ EpCAM+	2.25 ± 1.54 / 7.5mL	na	na	[132]
88		CellSearch System	Positive immuno-magnetic selection	DAPI+ CD45- CK+ EpCAM+	na	na	Tumor size, TNM stage, multi-nodularity, lymphatic invasion, mestasis, OS	[133]

Summary of CTC-studies conducted in PLC patients. Number of patients enrolled, platforms and methods used for CTC isolation and characterization, phenotype and number of isolated CTCs and correlation with clinical and pathological parameters or outcome of patient are reported, together with the relative references.

Furthermore, complexity of PLC-CSC heterogeneity can be deciphered in the context of stem-like plasticity [2, 47, 95]. Indeed during normal stem cell development, a continuum of stem/progenitor states implied a high degree of stemness variety [2, 42].

In this view, a better knowledge of liver CSC-biology and its role in human PLC-dissemination is essential to develop new molecular therapies effective in prolonging long-term survival in liver cancer patients [2, 47].

## METASTATIC CASCADE: EPITHELIAL-MESENCHYMAL PLASTICITY AND CIRCULATING TUMOR CELLS

Tumor metastasis represents a multistep process by which neoplastic cells escape the physical barriers at the primary site, enter the circulation, disseminate and proliferate into secondary sites [96, 97]. As a part of metastatic cascade, tumor cells lose their cell-to-cell adhesion and undergo EMT in order to enter bloodstream. Subsequently, the exit from circulation and generation of micrometastasis are both driven by the EMT-reverse program, the mesenchymal-epithelial transition (MET) [97–99]. Thus epithelial-mesenchymal plasticity is critical hallmark during the disseminating evolution suggesting that EMT drives tumor cell circulation while successive MET the metastatic colonization [96, 100, 101].

As tumor dissemination mainly occurs through the blood, the circulating tumor cells (CTCs) branch from tumor mass into the vasculature on their way to metastatic sites [99, 101–105]. The spread of CTCs released in the bloodstream from primary or metastatic tumors represents the major responsible for metastasis initiation and tumor recurrence [102, 106]. This highlights the reason why CTCs have recently emerged as potential novel biomarkers in oncological field.

According to several experimental and clinical studies, only a minim percentage of CTCs can survive in the bloodstream and within them a small subset is able to form macrometastasis in a diverse organ [96, 101, 103]. Therefore, metastatic-colonization is rather inefficient and not all primary tumor cells possess metastatic potential. Indeed, once in the bloodstream, CTCs meet three main obstacles to their survival and dissemination: 1) shear forces and collisions with leukocytes generated by blood flow, 2) defense activity of immune system, 3) absence of cell-matrix interactions that triggers an apoptotic process called anoikis. When CTCs overcome these impediments, they migrate into target tissue throw the association with platelets and endothelial cells lining the vessels [103]. However, during this step they can be entrapped in small capillaries, leading to the arrest of the disseminating process [96]. At the end, instead of total CTCs, less than 0,01% of CTCs are able to complete the late events of metastasis, representing the real initiating source of metastatic process [96, 99, 101, 103].

Several evidences suggest that distant organs are not casual target of metastatic process. Definitely, released signals from primary tumors (e.g. cytokines, exosomes, enzymes, etc.) are able to influence the microenvironment of target tissues, leading to the generation of a permissive pre-metastatic niche before CTC-extravasation [103]. Moreover, metastatic microenvironment may promote and support cancer cell dormancy, a state in which disseminated metastasis-initiating cells remain occult for an unlimited period of time [96, 99]. For this reason, patients may potentially remain in a clinical latency for years before appropriate microenvironmental signals re-activate proliferation of dormant tumor cells (DTCs) [96, 103]. Notably, cellular dormancy infers a quiescent non-proliferative state of tumor cells rendering them resistant to conventional therapies. Thus, targeting DTC-viability related-mechanisms might lead to eradication of residual disease and metastatic prevention [99, 103, 107].

## CTCS AND LIVER CANCER

High recurrence rate, most likely due to undetectable micrometastasis present at initial stage [108] is one of the principal causes of liver cancer death [109]. Investigation of PLC-CTCs is still at their very beginning in comparison with other tumor systems, particularly breast cancer. However recent evidence has revealed the importance of CTCs in liver carcinogenesis. Although CTCs represent a very challenging subset to be detected in peripheral blood (~1 CTC for every 10^7^ blood cells), analysis of circulating liver cancer cells is of fundamental relevance to provide safer, less painful, more accessible and dynamic information for recurrence and survival prediction [102, 106, 110, 111], thus highlighting its potential utility in PLC-surveillance. Instead of classical *in situ* biopsies and radiologic techniques, repeated collection of PLC-CTCs could be indeed used to monitor response to therapy [112–115], as already described in other solid tumors (e.g. melanoma [116], pancreatic [117], prostate [118, 119] breast [120–122], lung [123, 124] gastric [125] and colorectal cancer [126, 127]). Therefore CTC-analysis may be considered as ‘liquid biopsy’ with the theoretical advantage of serial sample collection and real time monitoring of disease progression [101, 108, 112, 128–130]. Indeed, molecular information derived from liquid biopsy could be potentially useful for a better understanding of molecular alterations that control tumor development and progression [129, 130].

**Figure 1:**
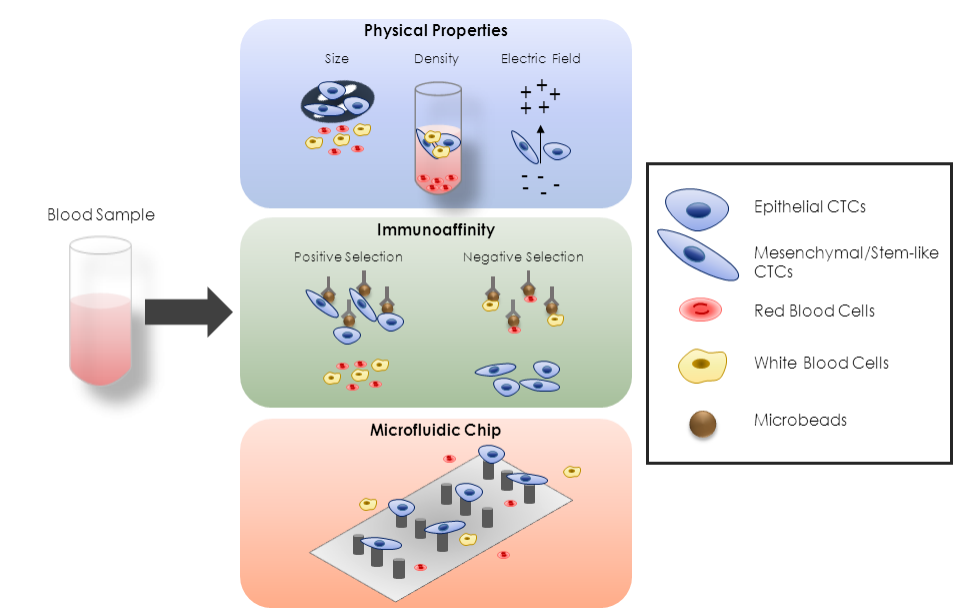
Different Approaches for CTC-Enrichment There are different conventional approaches for CTC-enrichment, based on diverse biological features. A first group is based on physical properties of CTCs, such as size, density and electric charge. The second group is based on immunoaffinity. In this case positive-selective techniques use microbeads targeting CTC-specific antigen, whereas microbeads targeting red or white blood cells are used in negative-selective strategies. The third group is represented by microfluidic-based platforms that incorporate a combination of size- and immunoaffinity-based approaches, thus representing a combination of the previous two groups.

Current methods for CTC-isolation and detection are described in BOX1 and summarized in Figure 1. In liver cancer, most evaluating CTC-studies widely employ immunoaffinity EpCAM-based methods [106, 108, 109, 131–133]. PLC-CTC number is very broad within a certain study and between the different studies reported below [53, 54, 108, 109, 112, 113, 115, 128, 131–138], likely due to the sample heterogeneity and technical platforms used for CTC-isolation.

### Evidence of CTCs in hepatocarcinoma

Although the hematogenous spread of CTCs from primary HCC is a crucial step in metastatic cascade, correlation of HCC progression and treatment-response to CTC-numbers remains to be elucidated.

*Xu et al.* measured CTCs in blood samples from 85 HCC patients at various stages and defined them as cell with larger cell size, intact nuclei, high nucleus-to-cytoplasm ratio, CD45- (leukocytes marker) and HepPar1+ (a hepatocyte-specific marker, which binds mitochondria antigens present in liver-derived cells). The authors showed that CTCs could be detected in 81% of HCC patients, even at early stage or with tumor size lesser than 2 cm, with a CTC-number ranged from 0 to 125 per 5 mL of blood, whereas no CTCs were identified in any of the healthy subjects’ blood samples. These data confirmed that tumor blood-borne dissemination could represent a very early event. Moreover, the positivity rate and the number of CTCs statistically correlated with portal vein tumor thrombus, tumor size, grading (defined by Edmondson-Steiner grading) and tumor-node-metastasis (TNM) staging. Interestingly, the positivity rate and number of CTCs was statistically higher in patients who did not meet the Milan criteria (commonly used to select patients eligible for liver transplantation) suggesting that CTC-amount could be better indicator of liver transplantation eligibility, thus directing the most suitable therapeutic intervention. Importantly, in order to minimize false-positive results during disease monitoring, the authors also observed that liver resection itself could cause hepatocyte release into the bloodstream that subsequently disappeared after 2 weeks in case of not cancerous cells [128].

Using a green fluorescent protein (GFP)-transfected HCC orthotopic mouse model, *Fan et al*. were able to continuously monitor CTC-dynamics by *in vivo* flow cytometry and confirmed that circulating HCC cells correlated with tumor size and metastasis. Although they demonstrated that liver resection might decrease CTCs at an undetectable level, a progressive CTC-enhancement could be detected when tumors were not completely removed [134], supporting the idea that CTCs may be a useful tool to monitor residual disease or presence of metastatic lesions.

Through a similar GFP-labeled HCC *in vivo* model, *Yan et al*. recently observed that, in response to Sorafenib treatment, a significant decrease in CTC-numbers and lung metastasis in addition to an increase in tumor necrosis and apoptosis as well as inhibition of tumor proliferation and angiogenesis [112].

Since CTC-presence in circulation may indicate tumor metastasis development, it might represent a helpful tool to guide treatments during neoadjuvant therapies before or after surgery. Importantly, not all HCC patients equally respond to Sorafenib. In this respect one of the reason might be found in the simultaneously inactivation of Ras/Raf/extracellular signal-regulated kinase (ERK) pathway and the activation of the phosphoinositide3-kinase (PI3K)/protein kinase B (Akt) / mammalian target of rapamycin (mTOR) in tumor cells as well as CTCs. Very recently, *Li et al*. evaluated the activation (phosphorylation) of ERK and Akt in CTCs obtained from 109 HCC patients. After two weeks of Sorafenib treatment, all patients showed a decline in CTC-number, but the highest decrease occurred in those with high proportion of pERK+/pAkt- CTCs, suggesting it as the most treatment-sensitive phenotype. In particular, in patient with < 40% proportion of pERK+/Akt- CTCs, Sorafenib-response correlated with a longer progression free survival and a better clinical outcome compared to patients with < 40% [113].

Additionally, *Sun et al*. detected EpCAM+ CTCs in 66.67% HCC patients and those with CTCs ≥ 2 per 7.5 mL (approximately 41%) showed higher tumor recurrence rate than patients with CTCs < 2 per 7.5 mL (70.6% *versus* 20.8%). Furthermore, EpCAM+ CTCs ≥ 2 were strongly associated with a shorter time of recurrence, greater vascular invasion, poor tumor differentiation and higher levels of alpha fetoprotein (AFP), an important HCC marker. Therefore the authors reported that determination of preoperative EpCAM+ CTC amount could be used as promising recurrence indicator [109].

In a pilot study *Kelley et al*. not only preceded to EpCAM+ CTCs detection and enumeration in 20 HCC patients, but more importantly they provided a CTC-characterization by next-generation sequencing. The authors identified about 58 somatic non-synonymous mutations in all CTC-samples, among which there were some liver emblematic mutations (e.g. *TP53, CTNNB1, PTEN, CDKN2A*), non-liver mutations (e.g. *ATM, BRAF, KIT*, members of *ERBB* and *FGFR* family) and completely novel variants (e.g. *NOTCH, CSF1R, KRAS, SMAD4*). Moreover, the presence of at least 1 CTC per 7.5mL of blood correlated with ≥ 400ng/mL and vascular invasion, confirming again the potential role of CTCs as biomarkers for HCC progression and invasion [131].

The statistically significant correlation of EpCAM+ CTCs with AFP ≥ 400ng/mL and vascular invasion was already been demonstrated in 2003 by *Schulze et al*. who, analyzing a total of 59 HCC patients and 19 controls, detected ≥ 1 per 7.5 mL in 30.5% of HCC group. The median overall survival was significantly shorter in CTC-positive patients (460 days *versus* 764 days) and a higher frequency of CTCs was detected in patients with an advanced HCC stage (defined by Barcelona Clinic Liver Cancer (BCLC) staging system; BCLC C) compared to those with local limited disease [108].

Additionally, *Guo et al.* proved that patients underwent tumor resection with persistent preoperative and postoperative presence of EpCAM mRNA+ exhibited shorter time to recurrence and higher recurrence rate. Moreover, the authors showed that EpCAM+ CTCs enriched by negative selection (CD45-) could have been used as alternative tool to monitor treatment-efficiency instead of standard radiologic follow-up, with the benefits of better safety avoiding the side effects associated with X-rays exposure [115].

Although, majority of these studies were based on EpCAM+ CTC-selection, *Li et al*. highlighted the significant combination with the expression of EMT-markers. Indeed, the authors were able to detect the presence of Twist (89.8%) or Vimentin (80.4%) positive CTCs in HCC patients. On the other hand, the co-expression of both markers was present in 69.6% of CTC-positive patients and was significantly associated with portal vein thrombus, TNM staging and tumor size, suggesting that identification of Vimentin/Twist+ CTCs in addition to EpCAM+ CTCs could provide a better tool in the valuation of HCC mestastasis and prognosis. Hence, in order to avoid CTC-underestimation, it is essential to optimize CTC-detection methods, by possibly including EMT-markers. The authors also highlighted the correlation between CTC-levels and HCC prognosis after liver tumor's resection or hepatic artery chemoembolization [135]. Unlike several studies in the context of liver resection, relationship between CTC-presence and HCC recurrence after liver transplantation (LT), has not been extensively described yet [136, 137]. Indeed further investigations of CTCs in LT clinical setting would certainly provide valuable information for prediction HCC prognosis after transplantation.

### Evidence of CTCs in cholangiocarcinoma

Dissimilar to hepatocarcinoma, very few CTC-studies have been performed in CCA underlining that development of his promising field is required.

First evidence dates back to 2008 when *Yang et al*. proved the existence of CD45- CD90+ CTCs in blood of patients with liver cancer, including CCA. Remarkably, this CTC-subset displayed tumor stem-like features, as evidenced by the expression of key stem-like genes including *Bmi1, CD44, Oct4, Notch1, Wnt3a, Stat3*, and *HIF-1α* [53, 54].

Later, *Al Ustwani et al*. tried to set a cut-off for positive CTC-value (2 CTCs/7.5 mL) in CCA with the use of CellSearch system and they identified presence of CTCs in 23% of CCA patients with a strong association with disease-stage. Moreover, at 12 months of follow-up, 25% of patients with positive CTC-value and 50% of those with negative CTC-rate were alive. Although no statistically significant correlation was found, a good trend between CTC-value and clinical outcome could be certainly observed. The inability to detect CTCs in a higher percentage of patients may found a possible explanation in the activation of EMT-program characterized by the loss of expression of epithelial antigens, such as EpCAM. Additionally, the limited number of patients enrolled in this study suggests that further deep investigation is required [132].

In a very latest work, the group of *Yang et al*. proceeded to CTC-enumeration in 88 CCA patients, among which 17% resulted positive for ≥ 2 and 9% for ≥5. Larger CTC-number appeared to correlate with a more extensive tumor burden, represented by larger tumor size, multi-nodularity, lymph node invasion and distant extrahepatic metastasis. Notably, a CTC-number^3^ ≥ 5 correlated with a shorter survival in intrahepatic CCA patients showing a trend toward its association with survival in non-metastatic CCA patients. Although this represents a pioneer study in CCA field, unfortunately several limitations are evident. First, CTC-detection relies on EpCAM expression that may be responsible of a possible CTC-underestimation. In addition, the entire study trusts on a very heterogeneous group of CCA patients in terms of CCA classification, presence/absence of metastasis and type of treatment, without focusing on specific CCA-subtypes (e.g. intrahepatic CCA, distal CCA and perihilar CCA or metastatic and non-metastatic CCA). Furthermore, since primary sclerosing cholangitis (PSC) represents a well-known risk factor for CCA-initiation, the study also included several PSC-associated CCA patients. Although, no detectable levels of CTCs were found, sample size was too small to stem solid conclusions [133, 138].

Hence, despite data on prognostic role of CTCs in CCA are very scarce, these studies suggest the importance of CTCs as a valuable tool in CCA clinical management.

In conclusion, similarly to other tumor models, the large amount of PLC-CTC studies allow the definition of key findings such as correlation with patients’ clinical and pathological data (tumor grade, TNM stage, recurrence, etc.) and treatment response, CTC-prognostic significance, CTC-heterogeneity as well as the inadequacy of EpCAM as only marker CTC-isolation and necessity to improve CTC-detection methods [53, 54, 108, 109, 112, 113, 115, 128, 131–138]. However, current PLC CTC-field requires to be further investigated.

## PHENOTYPICALLY DIVERSE CTC-SUBTYPES: STEM-LIKE PLASTICITY TRIGGERS A CONTINUUM OF EMT AND MET STATES

It's important to underline that not all CTCs are able to invade blood circulation [139] and only a small subset is capable to initiate secondary tumors [96, 99]. Mirroring complex heterogeneity of primary tumor cells, phenotypically distinct CTC-subtypes (including epithelial, hybrid epithelial-mesenchymal, mesenchymal and stem-like) expressing a variety of surface proteins with different behaviors (proliferation, cell cycle arrest, epithelial differentiation, dissemination) can co-exist in the circulation. These subpopulations are not strictly distinct as there is a continuum between their different stages [45, 104, 140, 141].

Indeed, epithelial CTCs maintain the expression of epithelial-specific markers, such as EpCAM and different type of cytokeratins such as CK8, CK18, CK19 thus retaining their original phenotype [106, 108, 109, 113, 131–133]. On the other hand, accumulating evidence shows that, as result of a complete EMT-adaptation of primary cancer cells, CTC-subsets retain a mesenchymal-like phenotype, characterized by an up-regulation of Vimentin and Ncadherin genes [45, 135, 142]. Because of EMT-process, tumor cells and derived-CTCs can undergo to various alterations during the early stages of carcinogenesis, leading to cancer cell dissemination and micrometastasis establishment. Indeed, mesenchymal phenotype of CTCs promotes migration and invasion, as well as the escape from immune surveillance, resistance to anoikis and shearing forces in the bloodstream [45, 101, 104, 135]. In this respect, EMT-positive CTCs could be considered as potential indicators of aggressive relapse and more importantly an essential tool for a better understanding of tumor recurrence.

In contrast, it has also been suggested that different CTC-subsets may be in transition from epithelial to mesenchymal state, retaining several intermediate phenotypes [101, 102, 104], as reported in patients with metastatic non-small-cell lung cancer (NSCLC) [143], early and metastatic breast cancer [144, 145] and prostate cancer [145]. *Nel et al*. in their study detected both epithelial (panCK+ and/or EpCAM+ cells) and mesenchymal (Vimentin+ or Ncadherin+ cells) CTCs in almost all enrolled HCC patients. Additionally only 36% of patients retained both epithelial and mesenchymal CTCs, underlining the concept that a variety of CTC-populations with different proportions and diverse roles could be identifiable in peripheral blood of tumor patients [146].

Instead of single cells, further complexity is described by the presence of CTCs as cluster (CTC-clusters) including relatively small entities (the majority) or dozens (the minority). At this regard, hybrid-EMT cells, preserving both epithelial (cell-cell adhesion) and mesenchymal (migration) features, are the ones who mostly undergo to a “co-migration” as CTC-clusters [98]. Interestingly, maintenance of a hypoxic microenvironment within CTC-cluster is essential to activate EMT-process thus rendering them much more resistant to apoptosis and tumorigenic as compared to single CTCs [142]. Therefore, size of CTC-clusters is indeed a critical issue. Overall, continuous presence of CTC-clusters in blood correlated with different levels of malignancy and metastatic potential as described in breast and prostate cancer patients with a dramatic shorter progression-free and overall survival, respectively [147]. In a recent work, *Aceto et al*. proved that CTC-clusters not represent an aggregation or a clonal progeny of individual CTCs into the vasculature, but more likely oligoclonal clumps of tumor cells within primary tumor mass. Although CTC-clusters represented only 2-5% of CTC-events in the circulation (likely due to their faster clearance rate as result of their entrapment in small capillaries), they possessed higher metastatic potential (about 23-50 times) than single CTCs. RNA sequencing of purified CTC-clusters permitted the identification of an important cell-junction component, plakoglobin, as a key orchestrator of CTC-cluster formation and stabilization, thus representing a potential target to reduce metastatic spread of breast cancer [147].

Only one small study reported the presence of CTC-clusters in HCC patients in which the authors detected mesenchymal phenotype of CTC-cluster in a liver cancer patient at T3N1M1 clinical stage [148]. With the exception of this analysis, presence of PLC-associated CTC-clusters has not been deeply evaluated yet.

**Figure 2:**
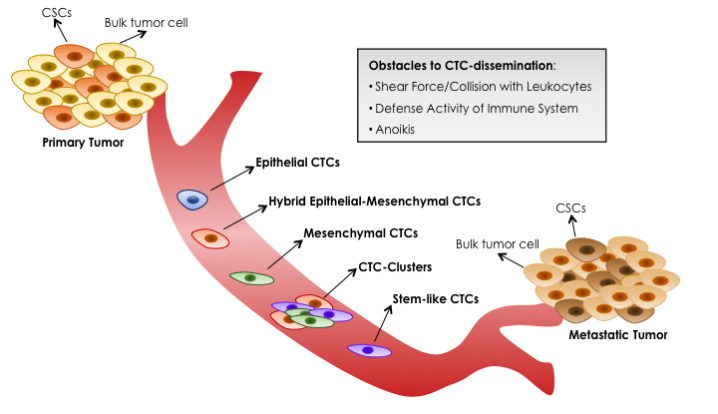
Blood Dissemination of Different CTC-subtypes Primary tumors, heterogeneously composed by bulk tumor cells and CSCs, can shed several CTCs, but only a minim percentage can survive in the bloodstream and only a minority is able to initiate metastatic tumor growth in a diverse organ. CTCs meet three main obstacles to their survival and dissemination: shear forces and collisions with leukocytes generated by blood flow, defense activity of immune system, absence of cell-matrix interactions that triggers an apoptotic process called anoikis. Mirroring complex heterogeneity of primary tumor cells, phenotypically distinct CTC-subtypes can co-exist in the circulation: epithelial, intermediate epithelial-mesenchymal, fully mesenchymal and stem-like CTCs. Moreover, CTCs with hybrid epithelial-mesenchymal phenotype can collectively migrate as CTC-clusters, instead of single cells. Emerging evidences suggest that only the subset of stem-like CTCs is endowed with ‘metastasis-initiating’ capability.

**Figure 3:**
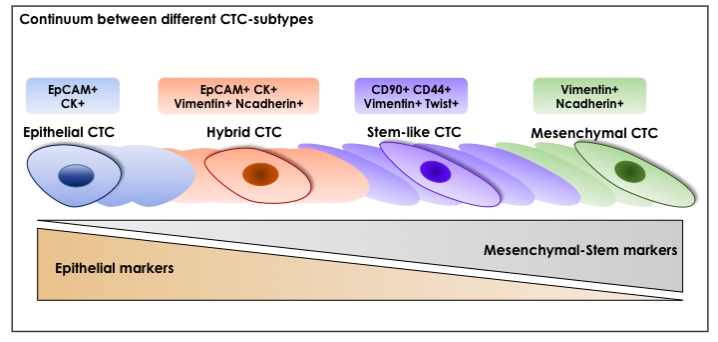
A Continuum Between Different CTC-Subtypes Among CTC-subpopulations, there are several phenotypes: epithelial CTCs that maintain the expression of original epithelial-specific markers (e.g. EpCAM and cytokeratins (CKs)); hybrid CTCs represented by cells in transition from epithelial to mesenchymal state, as demonstrating by the concomitant expression of both epithelial and mesenchymal markers; stem-like CTCs expressing both classical stem-like markers (such as CD90 and CD44) and mesenchymal-like markers (Vimentin and Twist); mesenchymal CTCs which show an upregulation of mesenchymal-like markers (such as Vimentin and Ncadherin). These subpopulations are not strictly distinct but a continuum between their different stages is probably existent.

Among the wide CTC-spectrum, the identification of stem-like “metastasis-initiating” CTC-subset may provide an attractive approach for both systemic cancer diagnosis and therapy. In view of tumor plasticity, the aforementioned EMT-program couples the two key concepts of metastatic-CTCs with relapse-initiating CSCs. In this regard, emerging evidences suggest that only a specific subset of CTCs with stem-like properties represents the ‘lethal seeds’ with superior adaptation under harshly adverse conditions of bloodstream as well as metastatic stroma where re-initiate growth and form metastases [96, 97, 99, 102–105]. Accordingly, with CSC-hypothesis, self-renewal ability and resistance to anti-cancer drugs make stem-like CTCs particularly hard to eradicate, leading to the permanence of minimal residual disease in cancer patients with a consequent cancer recurrence [140]. In this respect, analysis of stem-like CTCs is of fundamental relevance to provide unique molecular information about minimal residual disease in patients without clinically detectable metastatic lesions as well as to prevent tumor distant metastasis [110, 111]. In some cases, stem-like CTCs express both classical stem-like markers (e.g.CD44 [40, 44, 53, 54, 149–151], ABCG2 [109], ALDH1 [40, 44, 150, 151], DCLK1/Lgr5 [150], CD90 [54] and CD133 [109, 145]) as well as EMT-markers (e.g. Vimentin [109, 152], Twist [135]) reinforcing the concept that an aberrant activation of EMT-program enhances stemness properties and tumor-initiation potential of CTCs. Emerging evidence suggest that CTCs with hybrid or partial EMT-state, rather than those with a ‘fully’ epithelial or mesenchymal phenotype, retain stem-like features and are capable of completing the invasion-metastatic cascade. The presence of this great level of heterogeneity makes very challenging to find an accurate method for CTC-detection in blood patients. In order to target the diverse degree of EMT in the entire CTC-population, a combination of epithelial, mesenchymal and stem-related markers likely represent the ‘gold standard’ for CTC-enrichment [98]. A summary of different layers of CTC-heterogeneity is shown in Figure 2 and Figure 3.

## STEM-LIKE CTCS IN LIVER CANCER

One of the key problems in liver cancer treatment is the high recurrence rate, most probable due to presence of undetectable micrometastasis at initial stage [108]. Although multiple studies have demonstrated the existence of stem-like CTCs in HCC, a precise characterization of this highly aggressive population as well as the contribution of CSC to PLC -metastatic outgrowth need to be elucidated yet.

Based on finding in HCC tumor specimens, Yang *et al*. used CD90, a well-described liver CSC-related marker to characterize stem-like CTCs by investigation of CD45- CD90+ cells in blood samples of PLC patients. The authors identified CD45- CD90+ CTCs in 91% of samples and demonstrated that this cell population expressed key stem-like genes including *Bmi1, CD44, Oct4, Notch1, Wnt3a, Stat3*, and *HIF-1a* compared to CD90+ tumor-tissue cells. Moreover, CD45- CD90+ CTCs were more tumorigenic compared to CD45- CD90- population when orthotopically injected in the liver of severe-combined immunodeficient mice. Notably, a positive correlation between the number of CD45- CD90+ CTCs and tumor size was proved, whereas no association with disease free or overall survival was determined, probably due to the limited number of cases and short follow-up period. In addition, because of detectable levels of CD90+ CTCs in patients with small tumors (<5 cm) and dysplastic nodules, a fundamental role of this subset in early stage of hepatocarcinogenesis was revealed. All these findings suggested a potential implication of circulating stem-like HCC cells in disease surveillance [53, 54].

In a subsequent study, same authors detected CD45- CD90+ CD44+ CTCs in 68.3% of enrolled HCC patients, demonstrating a significant correlation with tumor size, TNM stage and, more relevantly, with post-hepatectomy recurrence and survival. Indeed, in presence of recurrence, average levels of CD45- CD90+ CD44+ CTCs were 0.02% as compared to 0.01% of non-recurrent patients. Moreover, the median recurrence-free survival (6 months) as well as overall survival (30 months) were lower compared to 46.5 months and 57.1 months of non-recurrent patients, respectively. Therefore, stem-like CTCs > 0.1% emerged to have a strong prediction ability of recurrence and survival, also much higher than conventional parameters, such as TNM staging, tumor size, vascular invasion [149].

Recently, *Zhu et al*. relying on the fact that stromal cell-derived factor-1 (SDF-1)/CXCR4 axis has a key function in regulation of cell migration and homing, hypothesized that CD90+ CXCR4+ cells could represent putative stem-like CTCs. Indeed, in only CD90+ CXCR4+ transplanted mice it was possible to detect tumor cells in circulation and tumor metastasis in distant organs thus suggesting that CD90+ CXCR4+ CTCs potentially retain stem-like metastasis initiating capability [139].

Although, all these studies suggested CD90 as a potential marker for circulating-CSC identification, several limitations such as its abundant expression also in normal hepatic stem/progenitors cells, in mesenchymal-like cells (e.g. fibroblasts) and vascular cells are present, thus indicating a lack of specificity [153].

Furthermore, *Sun et al*. suggested EpCAM as a reliable marker for stem-like CTCs, considering that it is typically co-expressed with other CSC-related markers, such as CD133 and ABCG2, or associated with β-catenin accumulation and Wnt pathway activation. Moreover, in some cases, patient derived EpCAM+ cells exhibited a mesenchymal phenotype (Vimentin+/Ecadherin-) and higher tumorigenic potential when injected in mice rather than the EpCAM- cells. The authors also provided an analysis of EpCAM+ CTC-count before and after curative surgery, subdividing patients according to alterations between preoperative and postoperative EpCAM+ CTC-levels using a cut off of 2 CTCs per 7.5 mL of blood. They demonstrated that patients with persistent preoperative and postoperative CTC count ≥ 2 were characterized by higher recurrence rate and shorter time to recurrence, underlining the prognostic significance of HCC CTC-positivity [109]. A limitation of this study is represented by the exclusive use of EpCAM marker for stem-like CTC-isolation. Indeed, considering the fact that CSC-features are often associated with EMT-traits with a consequent down-regulation of EpCAM expression, this only marker is not sufficient to identify liver stem-like CTCs.

For this reason, *Cheng et al*. proposed Lin28B, an oncofetal microRNA-binding protein with a role in regulating the expression of important pluripotent factors such as Oct4, Nanog and Sox2. Thus, the authors proceeded to the detection of Lin28B transcript by RT-PCR and found its expression in 33.3% of HCC cases, with a significant association with larger tumor size, higher tumor grade and earlier recurrence after hepatectomy. However, Lin28B mRNA was also detected in 5% of controls, thus indicating that further studies are surely needed to clarify its prognostic value. These findings importantly supported the redefinition of HCC staging system from TNM to TNMC (C for CTCs), accentuating the potential predicting value of CTCs [154].

On the other hand, relying on the expression of intercellular adhesion molecule 1 (ICAM1), *Liu et al*. in 2013 detected CD45- ICAM1+ cells in 60 patients with a frequency higher than 0.157% and a correlation with more aggressive tumor behavior and worse patients’ outcome. Moreover, CD45- ICAM1+ cells possessed *in vitro* stem-like properties, as demonstrated by sphere formation and *in vivo* tumor induction. Notably, ICAM1 inhibition by shRNA resulted in reduced tumor development and metastasis in mice, thus suggesting ICAM1 a good therapeutic target [155].

Strengthening the link between EMT-process and CTCs, a new mechanism for stem-like CTC-generation has been recently proposed. Indeed, it has been hypothesized that hepatic transmembrane 4 L six family member 5 (TM4SF5), which is overexpressed in HCC and implicated in EMT-program, may have a potential role in generating stem-like circulating HCC cells through the interaction with CD44 protein. After the binding of TM4SF5 to CD44, an activation of c-Src/STAT3/Twist1/Bmi1 signaling pathway promoted the release of metastatic stem-like CTCs in the blood of orthotopic mouse model. Interestingly, the knockdown of either TM4SF5 or CD44 or the disruption of their interaction abolished the presence of CTCs and metastatic properties [156].

Among all the studies presented here, the authors used different surface markers to define and detect stem-like CTCs in the blood of HCC patients, and in all cases, the identified subpopulations possess putative stem-like features. This may be attributable to the significant heterogeneity of CTCs, as already mentioned before.

Since only a minority of stem-like cells represent the driving force of tumor progression and metastasization, we can assert that the presence of CTCs itself is necessary but not sufficient for the initiation of metastasis. Hence, identifying the stem-like CTC-subset would provide more relevant prognostic information rather than total CTC-counts. At this regard, few studies emphasized the prognostic potential of stem-like CTC-levels in patients underwent curative resection, underlining the correlation between persistent pre- and post-operative presence of metastasis-initiating CTC-subset together with lower relapse-free survival [109, 157].

## CONCLUSIONS AND FUTURE DIRECTIONS

There is general consensus in cancer research field regarding intriguing biological features and clinical significances of stem-like cells within single tumors. Due to its key role in tumor recurrence and metastasis spread, they represent a therapeutic challenge subpopulation. Thus, supposing that CSCs account for tumor metastasis, stemness features should be found among tumor cells disseminated from primary tumors including CTCs. Although it has been shown that activation of EMT-program, by generating a subpopulation endowed with stem-like traits capable of self-renewal and drug-resistance, represents a central biological link between CSCs and CTCs, many ambiguities do exist and a solid molecular signature which confirmed their correlation still need to be established.

Indeed, central debate aims to understand whether tumor cells undergoing EMT-process result in a more mesenchymal or even more stem cell-like phenotype and what fraction of CTCs has better metastatic seeding potential or increased therapeutic resistance.

Moreover, similar to normal stem cells and because of dynamic state of CSCs, a continuum of phenotypically distinct CTC-subtypes ranging from epithelial, hybrid epithelia-mesenchymal, mesenchymal and stem-like may co-exist in the circulation, reinforcing the concept of tumor heterogeneity (Figure 2, 3).

It is important to note that, CTC-detection remains technically challenging due to very low CTC-concentrations (one tumor cell against millions of blood cells). Thus, their identification and characterization require extremely sensitive and specific methods, which are usually represented by a combination of multiple procedures. Moreover, current PLC-CTC detection methods use the epithelial marker EpCAM, which may underestimate CTC-numbers and potentially fail to detect a critical subpopulation. If only CTCs that undergo EMT are those with stem-like features, then a combination of epithelial, mesenchymal and stem-related markers should be included. Because not all detected CTCs have metastatic and relapsing potential, a simple enumeration of CTCs without molecular characterization may lead to wrongful clinical conclusions. Elucidating CTC-biology at molecular level based on genomic profile would improve detection and isolation of the potentially disseminating CTC-subsets. In this regard, single-cell transcriptomic analyses of individual PLC-CTCs would benefit to guide clinical treatments and achieve much improved cure rate in liver cancer patients.

Exploring the existence of disseminating stem-like CTCs and their unique ability to initiate relapse and propagate metastatic growth, is essential to move to effective personalized therapy with the overall objective of identifying prognostic and diagnostic markers as well as novel therapeutic targets. Therefore, it would be pivotal to rule out a possible recurrence and metastatic dissemination at the time of diagnosis, by investigation of stem-like metastasis-initiating CTCs in order to discriminate high-risk factors of PLC recurrent patients after curative resection as well as to identify new therapeutic agents by targeting liver stem-like circulating cancer cells.

## BOX 1

### Conventional approaches for CTC-enrichment

Since approximately less than 1 CTC is present in 10^7^ blood cells, they represent a very challenging subset to detect [110, 111].

In recent years, many different CTC-isolation technologies have emerged to improve CTC-enrichment rates from other blood components [109, 165–171]. Based on diverse CTC-features, most approaches relied on 1) physical properties, 2) presence of specific cell markers and 3) combination of both. A summary of diverse methodologies is shown in Figure 1.

#### Methods based on physical properties

Strategies for CTC-enrichment centered on biophysical properties have gained increasing popularity and rely on the ability to discriminate between CTCs and other cells (e.g. leukocytes) based on physical characteristics such as size, density and electric charge [172] (Figure 1).

Several studies of CTC-physical properties revealed that these heterogeneous cells are typically larger (12-25 μm) than other white blood cells (8-14 μm) [173–177]. Two commercial size-based filtration platforms, ISET (Rarecells Diagnostics) and ScreenCell (ScreenCell), have been used to enrich CTCs based on their larger morphology compared to leukocytes. For instance, ISET, or ‘isolation by size of epithelial tumor cells’ was used to enrich fixed CTCs from blood samples through 8-mm pores polycarbonate [178].

Density gradient centrifugation is a conventional approach for separating blood components because of differences in their sedimentation coefficients. Indeed CTCs have a specific gravity (1.056) compared to red blood cells (1.092) and leukocytes (1.065). Although not originally developed for CTC-isolation, researchers have used Ficoll-Paque in this application and depending on their density, cells distribute along the gradient after centrifugation and erythrocytes or polymorphonuclear leukocytes migrate to the bottom, whereas mononuclear leukocytes and CTCs remain at the top [175, 179]. In addition to density gradient separation, OncoQuick system (Greiner Bio-one) incorporates a porous membrane above the separation media, which captures CTCs while allowing contaminating blood cells with similar densities to CTCs [175].

Dielectrophoresis (DEP) based method, relies on diverse electrical properties of tumor cells, which depend on composition (e.g. cell membrane, nucleus, organelles), morphology (e.g. size, shape), and phenotype [180, 181]. During DEP isolation, an attractive or repulsive force is exerted on a cell causing it to move towards or away from the electrical field source. The commercial technology DEPArray (Silicon Biosystems) traps single cells in DEP cages generated via an array of individually controllable electrodes [182–187].

These approaches allow viable CTC-isolation from blood avoiding the use of tumor markers. On the other side, cancer patient blood may contain a mixture of diverse cells deriving from organs which are not necessary tumor cells. Moreover, CTCs may retain larger size than leukocytes making them not suitable for ISET or ScreenCell filters. Even if tumor cells are captured on the membrane, it may be difficult to detach them for further molecular examination. Density gradient centrifugation may instead lead to a loss of tumor cells resulting in false-negative results.

#### Methods based on immunoaffinity

The second group mainly relies on the use of antibodies against tumor specific biomarkers (Figure 1). Indeed, immunoaffinity-based approaches targeting EpCAM is the most used technique for positive selection of CTCs because its expression is virtually universal (albeit at variable levels) in cells of epithelial origin and absent in blood cells [109, 166].

Alternative CTC-isolation platforms deploy a strategy of negative selection (such as for CD45- cells) to remove mononuclear cells and primarily leukocytes [109, 188].

Other isolation platforms have developed magnetic microbeads coated with antibodies against CTC cell-surface proteins such as EpCAM. These include a magnet-activated cell sorting system (MACS, Miltenyi) and Dynabeads (Invitrogen) that provide a release buffer to remove the beads from cells [188, 189]. Recently, the AdnaTest platform (AdnaGen GmbH) combines several antibodies specific to unique cancer types, with reverse transcription-PCR and multiplex PCR to analyze CTC-expression of tumor markers [190].

Nevertheless, the only clinically validated FDA-approved test to capture and enumerate CTCs is represented by Cell Search^®^ system. In this platform, tumor cells are first immunomagnetically enriched by EpCAM antibody-coupled magnetic beads. Subsequently, EpCAM positively recovered cells are recognized for negative CD45 staining, positive DAPI nuclear content and cytoplasmic epithelial markers cytokeratin (CK) 8, CK18, CK19 [167].

This second group of methods provides alive epithelial cells, which may be used for further analyses as well as *in vitro* growth. However, although immune-enrichment methods could acquire high CTC-purity, on the other hand they might miss the highly metastatic tumor cells which, due to the EMT-process, are characterized by decreased expression of epithelial markers and the acquisition of mesenchymal features [168]. Therefore, alternative enrichment approaches should also consider the combination with mesenchymal markers. Since EpCAM and CKs are also expressed by circulating epithelial non-tumor cells, thus they are not CTC-specific and can lead to false positives. Additionally, purity-related issues may arise due to non-specific binding of cells to the microbeads. These disadvantages are mostly problematic in the clinical setting.

#### Combinatorial methods

Most recently, the microfluidic-based “CTC-chip” has attracted intense attention due to its enhanced sensitivity and specificity in CTC-purification [169, 170] (Figure 1). This third group allows precise control of tiny volumes of fluids under flow through antibody-coated microspots providing a functionalized surface and dynamic flows that exclude nonspecific binding [171]. Although such platforms have increased the sensitivity of CTC-detection and capture, their low flow rates don't allow to process sufficient volume sample for a high CTC-yield.

Several microfluidic-based platforms have more recently incorporated a combination of size- and immunoaffinity-based approaches to develop integrated systems and improve CTC-recovery [147, 191–194]. *Ozkumur et al*. described the microfluidic CTC iChip, which combines hydrodynamic sorting, inertial focusing, and magnetic sorting of pre-labeled CTCs from blood [195]. An alternative microfluidic approach involves the use of microtraps which take advantage of CTC-size and deformability of [196].

Major challenges for these new techniques include cell fragility, blood tendency to clog, and high blood cellularity. Relevant parameters are sensitivity, purity (depending on leucocytes contamination rate), time for isolation, quality of cell morphology and cell structure for further morpho-immuno-molecular analyses, and viability for culture tests.

## KEY POINTS

- Poor prognosis and high recurrence represent leading causes of primary liver cancer mortality
- Spread of circulating tumor cells (CTCs) in the blood accounts for tumor recurrence and metastasis initiation
- CTCs can be considered an useful tool to monitor early blood dissemination of liver tumor cells and predict liver cancer progression, prognosis and therapy response
- CTCs are likely heterogeneous and only a subset of CTCs can survive in the bloodstream, migrate to distant sites and establish secondary tumors. Consistent with cancer stem cell (CSC) hypothesis, stem-like CTCs might represent a potential source for cancer relapse and metastasis
- Aberrant activation of a latent embryonic program (known as the epithelial-mesenchymal transition, EMT) generates undifferentiated cancer cells endow with stemness traits, thus providing a ready source of CSC-state. Although it might represent a crucial biological link between CSCs and CTCs, many ambiguities do exist and a solid molecular signature that confirmed their correlation still need to be established
- Similar to normal stem cells and as a consequence of dynamic state of CSCs, a continuum of phenotypically distinct CTC-subtypes ranging from epithelial, partial-EMT, mesenchymal and stem-like may co-exist in the circulation, reinforcing the concept of tumor heterogeneity
- Identification of stem-like metastasis-initiating CTC-subset may provide valuable clinically prognostic information in the context of personalized care management
